# Targeting Oxidative Stress for Disease

**DOI:** 10.3390/ijms26062692

**Published:** 2025-03-17

**Authors:** Nada Oršolić, Maja Jazvinšćak Jembrek

**Affiliations:** 1 Division of Animal Physiology, Faculty of Science, University of Zagreb, Rooseveltov trg 6, HR-10000 Zagreb, Croatia; 2 Croatia Division of Molecular Medicine, Laboratory for Protein Dynamics, Ruđer Bošković Institute, Bijenička Cesta 54, HR-10000 Zagreb, Croatia; maja.jazvinscak.jembrek@irb.hr; 3 School of Medicine, Catholic University of Croatia, Ilica 244, HR-10000 Zagreb, Croatia

Oxidative stress (OS) refers to a metabolic imbalance caused by the excessive production of reactive oxygen species (ROS) and an insufficient antioxidant defense. It plays a key role in the development of numerous diseases, including neurological disorders, type 2 diabetes, cancer, aging, heart and acute renal failure, hypertension, preeclampsia, atherosclerosis, coronary artery disease, asthma, chronic obstructive pulmonary disease, rheumatoid arthritis, glaucoma, osteoporosis, and sexual dysfunction [1,2,3,4]. Therefore, this Special Issue, title “Targeting Oxidative Stress for Disease”, aimed to provide recent insights into the role of ROS-mediated effects in both physiological and pathological processes underlying disease development.

OS is most commonly associated with neurological disorders. Increasing evidence suggests that OS plays a role in neurodevelopmental disorders, being one of the major environmental factors driving genomic instability and the deregulation of gene expression. Mahmoudi and co-authors [5] analyzed the expression of circular RNAs (circRNAs), a class of non-coding RNAs, and their role in competitive endogenous (ceRNA) networks (including circRNA, miRNA, and mRNA) in differentiating SH-SY5Y neuroblastoma cells that are exposed to OS before and during differentiation. The circRNAs containing miRNA recognition elements are of particular interest, because they act as miRNA sponges. By sequestering miRNAs, circRNAs prevent them from binding to their target mRNAs, thereby influencing various cellular processes, including gene expression [6]. Research on SH-SY5Y cells has shown that circRNAomes’ response to OS differs between early neural progenitor cells and cells undergoing neuronal differentiation, with the early stages being the more sensitive to OS exposure. These findings offer valuable insights into the regulatory mechanisms that are induced by OS and other environmental factors that may influence neuronal development [6].

OS and reduced antioxidant capacity have been implicated in the pathogenesis of autism spectrum disorder (ASD), a neurodevelopmental disorder with an increasing global incidence. While the causes of ASD are rather complex and not fully understood, OS biomarkers have been proposed as a potential approach for early diagnosis and the evaluation of pharmacological and nutritional treatments [7]. Moreover, antioxidant supplementation has been considered as a potential strategy for improving metabolic imbalances in ASD [8]. Interestingly, at the molecular level, ASD subtypes may be distinguished based on the ferroptosis score and expression of selected ferroptosis-related genes [9]. Ferroptosis, a form of cell death that is triggered by an Fe^2+^ overload and excessive ROS accumulation, has been linked to the pathophysiology of ASD. In animal models, attenuating ferroptosis has been shown to mitigate autism-like behaviors, further supporting the potential clinical application of OS-targeting strategies [10]. ASD is also linked with inflammatory abnormalities, and treatment with anti-inflammatory agents may be beneficial for some individuals. However, further randomized placebo-controlled trials are required to determine the effectiveness of immunoregulatory agents as treatment options for ASD patients [11,12]. The anti-epileptogenic potential of certain compounds may also rely on their ROS scavenging capabilities [13]. Psychiatric disorders are similarly influenced by OS. A better understanding of the role of OS in the development and progression of psychiatric diseases could lead to novel therapeutic approaches aimed at modifying the severity of OS. However, the effects of neuropsychiatric drugs on OS are contradictory: some exhibit protective effects, while others disrupt redox homeostasis [14,15,16]. Therefore, in order to improve the clinical outcomes, future research should focus on rigorous clinical trials to clarify how different neuropsychiatric drugs modulate OS in distinct patient subgroups.

Antioxidants may also play a beneficial role in bronchopulmonary dysplasia, the most common respiratory complication in premature infants [17]. Although the pathogenesis of this disease is rather complex, it is mainly related to OS due to the limited antioxidant capacity of premature infants, along with inflammatory complications [18]. Emerging evidence suggests that next-generation antioxidants, which combine antioxidant and anti-inflammatory properties, hold promise for preventing and treating this condition [19,20]. The potential of antioxidants has also been explored for the treatment of cardiovascular complications of cirrhosis [21].

The potential of OS has also been studied in the context of cancer treatment. Although further research is needed to fully elucidate the contribution of OS to the development, progression, and treatment outcomes of cancer, an increasing amount of evidence suggests that ROS enhancement correlates with drug resistance, tumor aggressiveness, and poorer survival rates [22,23]. Cancer cells undergo redox reprogramming by simultaneously increasing ROS production and antioxidant defenses, optimizing ROS-driven proliferative signals while minimizing oxidative damage to intracellular targets [24]. Moreover, an ROS increase typically creates an inflammatory environment that promotes tumor growth and metastatic potential [25]. Despite significant advances in cancer treatment, drug resistance remains a major challenge. Therefore, novel chemopreventive agents are urgently needed to improve the overall efficiency of existing treatment options. By their ability to detoxify ROS, increased levels of various antioxidant enzymes may interfere with the efficacy of various anti-cancer therapies that rely on ROS-mediated cytotoxicity. Consequently, inhibitors of antioxidant enzymes and other antioxidant defense mechanisms have emerged as potential strategies for the improvement of anti-cancer therapies and overcoming treatment resistance [26,27,28,29].

Dietary approaches may also be effective in cancer prevention and treatment [29,30]. Various phytochemicals and bioactive natural products, such as royal jelly or propolis, show anti-inflammatory and anti-proliferative activities, acting synergistically with anti-cancer drugs to enhance the treatment efficacy and reduce side effects. In addition, these multitarget compounds may regulate the signaling pathways, antioxidant levels, and genetic and epigenetic instability that promote tumor growth [31,32,33]. However, despite the extensive preclinical evidence supporting the therapeutic potential of natural compounds, future research should prioritize clinical trials to determine their exact role as complementary cancer treatments.

In conclusion, OS contributes to the pathology of numerous diseases. While antioxidants appear to be a rational strategy for preventing and treating OS-related conditions, clinical trial results have been inconsistent. Hopefully, the data presented in this Special Issue should be an incentive for future clinical research that will advance our understanding of the role of OS and the therapeutic potential of OS-targeted therapies for improving human health and well-being.

## References

[1] Forman H.J., Zhang H. (2021). Targeting oxidative stress in disease: Promise and limitations of antioxidant therapy. Nat. Rev. Drug Discov..

[2] Reddy V.P. (2023). Oxidative Stress in Health and Disease. Biomedicines.

[3] Vona R., Pallotta L., Cappelletti M., Severi C., Matarrese P. (2021). The Impact of Oxidative Stress in Human Pathology: Focus on Gastrointestinal Disorders. Antioxidants.

[4] Jomova K., Raptova R., Alomar S.Y., Alwasel S.H., Nepovimova E., Kuca K., Valko M. (2023). Reactive oxygen species, toxicity, oxidative stress, and antioxidants: Chronic diseases and aging. Arch. Toxicol..

[5] Mahmoudi E., Khavari B., Cairns M.J. (2024). Oxidative Stress-Associated Alteration of circRNA and Their ceRNA Network in Differentiating Neuroblasts. Int. J. Mol. Sci..

[6] Hansen T.B., Jensen T.I., Clausen B.H., Bramsen J.B., Finsen B., Damgaard C.K., Kjems J. (2013). Natural RNA circles function as efficient microRNA sponges. Nature.

[7] Liu X., Lin J., Zhang H., Khan N.U., Zhang J., Tang X., Cao X., Shen L. (2022). Oxidative Stress in Autism Spectrum Disorder-Current Progress of Mechanisms and Biomarkers. Front. Psychiatry.

[8] Manivasagam T., Arunadevi S., Essa M.M., SaravanaBabu C., Borah A., Thenmozhi A.J., Qoronfleh M.W. (2020). Role of Oxidative Stress and Antioxidants in Autism. Adv. Neurobiol..

[9] Liu L., Lai Y., Zhan Z., Fu Q., Jiang Y. (2022). Identification of Ferroptosis-Related Molecular Clusters and Immune Characterization in Autism Spectrum Disorder. Front Genet..

[10] Wu H., Luan Y., Wang H., Zhang P., Liu S., Wang P., Cao Y., Sun H., Wu L. (2022). Selenium inhibits ferroptosis and ameliorates autistic-like behaviors of BTBR mice by regulating the Nrf2/GPx4 pathway. Brain Res. Bull..

[11] Arteaga-Henríquez G., Gisbert L., Ramos-Quiroga J.A. (2023). Immunoregulatory and/or Anti-inflammatory Agents for the Management of Core and Associated Symptoms in Individuals with Autism Spectrum Disorder: A Narrative Review of Randomized, Placebo-Controlled Trials. CNS Drugs.

[12] Jyonouchi H. (2024). Autism spectrum disorder and a possible role of anti-inflammatory treatments: Experience in the pediatric allergy/immunology clinic. Front. Psychiatry.

[13] Borowicz-Reutt K.K., Czuczwar S.J. (2020). Role of oxidative stress in epileptogenesis and potential implications for therapy. Pharmacol. Rep..

[14] Behr G.A., Moreira J.C., Frey B.N. (2012). Preclinical and clinical evidence of antioxidant effects of antidepressant agents: Implications for the pathophysiology of major depressive disorder. Oxidative Med. Cell Longev..

[15] Caruso G., Grasso M., Fidilio A., Torrisi S.A., Musso N., Geraci F., Tropea M.R., Privitera A., Tascedda F., Puzzo D. (2021). Antioxidant Activity of Fluoxetine and Vortioxetine in a Non-Transgenic Animal Model of Alzheimer's Disease. Front. Pharmacol..

[16] Yang M., Wang C., Zhao G., Kong D., Liu L., Yuan S., Chen W., Feng C., Li Z. (2023). Comparative Analysis of the Pre- and Post-Medication Effects of Antipsychotic Agents on the Blood-Based Oxidative Stress Biomarkers in Patients with Schizophrenia: A Meta-Analysis. Curr. Neuropharmacol..

[17] Perrone S., Tataranno M.L., Buonocore G. (2012). Oxidative stress and bronchopulmonary dysplasia. J. Clin. Neonatol..

[18] Saugstad O.D. (2003). Bronchopulmonary dysplasia-oxidative stress and antioxidants. Semin. Neonatol..

[19] Ferrante G., Montante C., Notarbartolo V., Giuffrè M. (2022). Antioxidants: Role the in prevention and treatment of bronchopulmonary dysplasia. Paediatr. Respir. Rev..

[20] Teng M., Wu T.-J., Jing X., Day B.W., Pritchard K.A., Naylor S., Teng R.-J. (2024). Temporal Dynamics of Oxidative Stress and Inflammation in Bronchopulmonary Dysplasia. Int. J. Mol. Sci..

[21] Ferlitsch A., Pleiner J., Mittermayer F., Schaller G., Homoncik M., Peck-Radosavljevic M., Wolzt M. (2005). Vasoconstrictor hyporeactivity can be reversed by antioxidants in patients with advanced alcoholic cirrhosis of the liver and ascites. Crit. Care Med..

[22] Oshi M., Gandhi S., Yan L., Tokumaru Y., Wu R., Yamada A., Matsuyama R., Endo I., Takabe K. (2022). Abundance of reactive oxygen species (ROS) is associated with tumor aggressiveness, immune response, and worse survival in breast cancer. Breast Cancer Res. Treat..

[23] Zhou X., An B., Lin Y., Ni Y., Zhao X., Liang X. (2023). Molecular mechanisms of ROS-modulated cancer chemoresistance and therapeutic strategies. Biomed. Pharmacother..

[24] Gu X., Mu C., Zheng R., Zhang Z., Zhang Q., Liang T. (2024). The Cancer Antioxidant Regulation System in Therapeutic Resistance. Antioxidants.

[25] Yu W., Tu Y., Long Z., Liu J., Kong D., Peng J., Wu H., Zheng G., Zhao J., Chen Y. (2022). Reactive Oxygen Species Bridge the Gap between Chronic Inflammation and Tumor Development. Oxidative Med. Cell. Longev..

[26] Yang H., Villani R.M., Wang H., Simpson M.J., Roberts M.S., Tang M., Liang X. (2018). The role of cellular reactive oxygen species in cancer chemotherapy. J. Exp. Clin. Cancer Res..

[27] Udomsak W., Kucinska M., Pospieszna J., Dams-Kozlowska H., Chatuphonprasert W., Murias M. (2024). Antioxidant Enzymes in Cancer Cells: Their Role in Photodynamic Therapy Resistance and Potential as Targets for Improved Treatment Outcomes. Int. J. Mol. Sci..

[28] Kong Q., Lillehei K.O. (1998). Antioxidant inhibitors for cancer therapy. Med. Hypotheses.

[29] Sznarkowska A., Kostecka A., Meller K., Bielawski K.P. (2017). Inhibition of cancer antioxidant defense by natural compounds. Oncotarget.

[30] Choudhari A.S., Mandave P.C., Deshpande M., Ranjekar P., Prakash O. (2020). Phytochemicals in Cancer Treatment: From Preclinical Studies to Clinical Practice. Front. Pharmacol..

[31] Lyubitelev A., Studitsky V. (2023). Inhibition of Cancer Development by Natural Plant Polyphenols: Molecular Mechanisms. Int. J. Mol. Sci..

[32] Salama S., Shou Q., Abd El-Wahed A.A., Elias N., Xiao J., Swillam A., Umair M., Guo Z., Daglia M., Wang K. (2022). Royal Jelly: Beneficial Properties and Synergistic Effects with Chemotherapeutic Drugs with Particular Emphasis in Anticancer Strategies. Nutrients.

[33] Oršolić N., Jazvinšćak Jembrek M. (2022). Molecular and Cellular Mechanisms of Propolis and Its Polyphenolic Compounds against Cancer. Int. J. Mol. Sci..

